# Author Correction: Structure and function of a β-1,2-galactosidase from Bacteroides *xylanisolvens*, an intestinal bacterium

**DOI:** 10.1038/s42003-025-07620-z

**Published:** 2025-02-07

**Authors:** Yutaka Nakazawa, Masumi Kageyama, Tomohiko Matsuzawa, Ziqin Liang, Kaito Kobayashi, Hisaka Shimizu, Kazuki Maeda, Miho Masuhiro, Sei Motouchi, Saika Kumano, Nobukiyo Tanaka, Kouji Kuramochi, Hiroyuki Nakai, Hayao Taguchi, Masahiro Nakajima

**Affiliations:** 1 Department of Applied Biological Science, Faculty of Science and Technology, Tokyo University of Science, 2641 Yamazaki, Noda, Chiba 278-8510 Japan; 2 Department of Applied Biological Science, Faculty of Agriculture, Kagawa University, 2393 Ikenobe, Miki, Kagawa 761-0795 Japan; 3 Artificial Intelligence Research Center, National Institute of Advanced Industrial Science and Technology (AIST), 2-4-7 Aomi, Tokyo, Koto-ku 135-0064 Japan; 4 Faculty of Agriculture, Niigata University, 8050 Ikarashi 2-no-cho, Niigata, Nishi-ku 950-2181 Japan

**Keywords:** Glycobiology, Hydrolases, X-ray crystallography

Correction to: *Communications Biology* 10.1038/s42003-025-07494-1, published online 16 January 2025

In the version of the article initially published, there was an error in the chemical structure of β-1,2-galactooligosaccharide in the graphical abstract, where oxygen atoms were missing from the pyranose rings. The error has been corrected in the HTML and PDF versions of the article.

