# Supplementary material for: Structure and function of a β-1,2-galactosidase from Bacteroides xylanisolvens, an intestinal bacterium
Source: Commun Biol. 2025 Jan 16;8:66. doi: 10.1038/s42003-025-07494-1 (PMC11739564; doi:10.1038/s42003-025-07494-1)
Supplement: Supplementary file 1 — Supplementary information [file 42003_2025_7494_MOESM1_ESM.pdf]

Supplemental information

Structure and function of a β-1,2-galactosidase from *Bacteroides xylanisolvens*, an intestinal bacterium

Yutaka Nakazawa[1], Masumi Kageyama[1], Tomohiko Matsuzawa[2], Ziqin Liang[1], Kaito Kobayashi[1,3], Hisaka Shimizu[1], Kazuki Maeda[1], Miho Masuhiro[1], Sei Motouchi[1], Saika Kumano[1], Nobukiyo Tanaka[1], Kouji Kuramochi[1], Hiroyuki Nakai[4], Hayao Taguchi[1], Masahiro Nakajima[1,*]

[1] Department of Applied Biological Science, Faculty of Science and Technology, Tokyo University of Science, 2641 Yamazaki, Noda Chiba, 278-8510, Japan

[2] Department of Applied Biological Science, Faculty of Agriculture, Kagawa University, 2393 Ikenobe, Miki, Kagawa, 761-0795, Japan

[3] Artificial Intelligence Research Center, National Institute of Advanced Industrial Science and Technology (AIST), 2-4-7 Aomi, Koto-ku, Tokyo, 135-0064, Japan

[4] Faculty of Agriculture, Niigata University, 8050 Ikarashi 2-no-cho, Nishi-ku, Niigata, 950-2181, Japan

*Corresponding author

m-nakajima@rs.tus.ac.jp; Tel, +81-471-24-1501

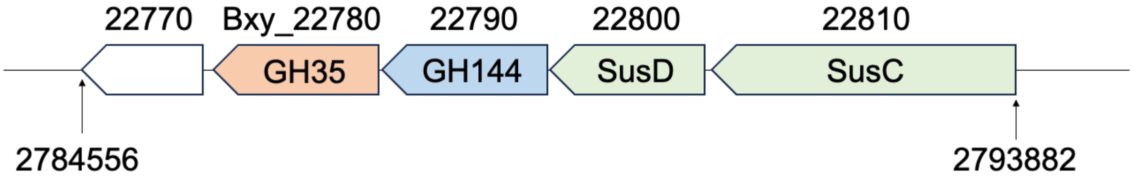

Supplementary Figure 1. The gene cluster around the gene encoding GH144 enzyme in *B. xylanisolvens* genome.

Genes and their directions are shown by arrows. Annotation of functions of the genes are represented by color in the arrows: GH35, light orange; GH144, light blue; SusCD, light green. The gene loci (KEGG database, https://www.genome.jp/kegg/) are shown above the arrows, and "Bxy_" are omitted except Bxy_22780. The numbers below the genes are nucleotide numbers from 5′ of the genomic DNA.

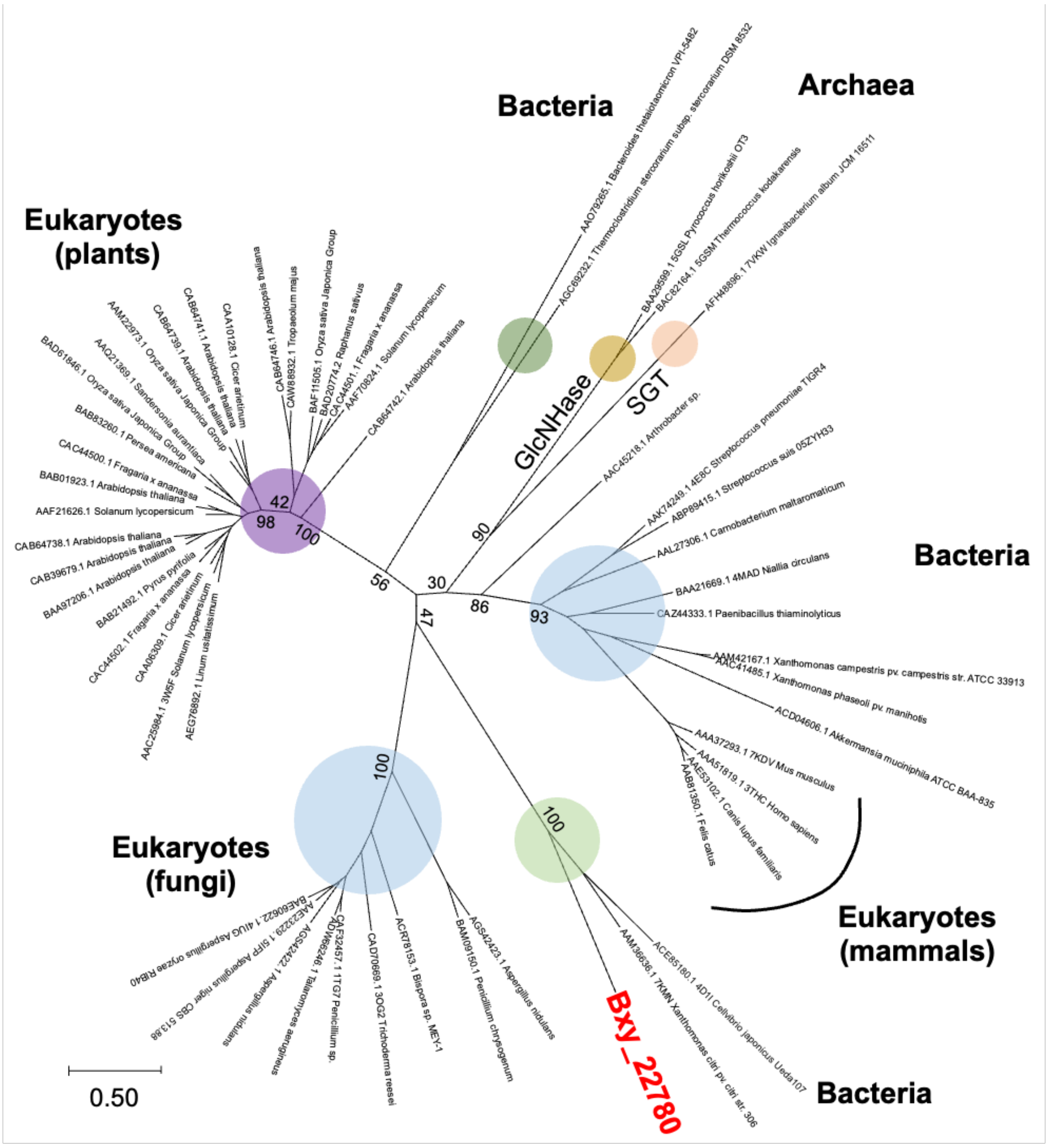

Supplementary Figure 2. Phylogenetic tree of GH35 enzymes.

Bxy_22780 is shown in red. The other proteins are indicated with GenBank accession No., organism names, and PDB IDs (if available). GlcNHase and SGT are abbreviations of β-glucosaminidase and β-1,2-glucosyltransferase, respectively. All except GlcNHases and SGT (enzymes without enzyme names) are β-galactosidases. A scale bar is shown at bottom left of the figure. Phylogenetic groups are visualized by colored circles. The phylogenetic tree was prepared by MEGA X [1] using maximum likelihood method with 1000 bootstrap replicates. Bootstrap values are shown at the major branches.

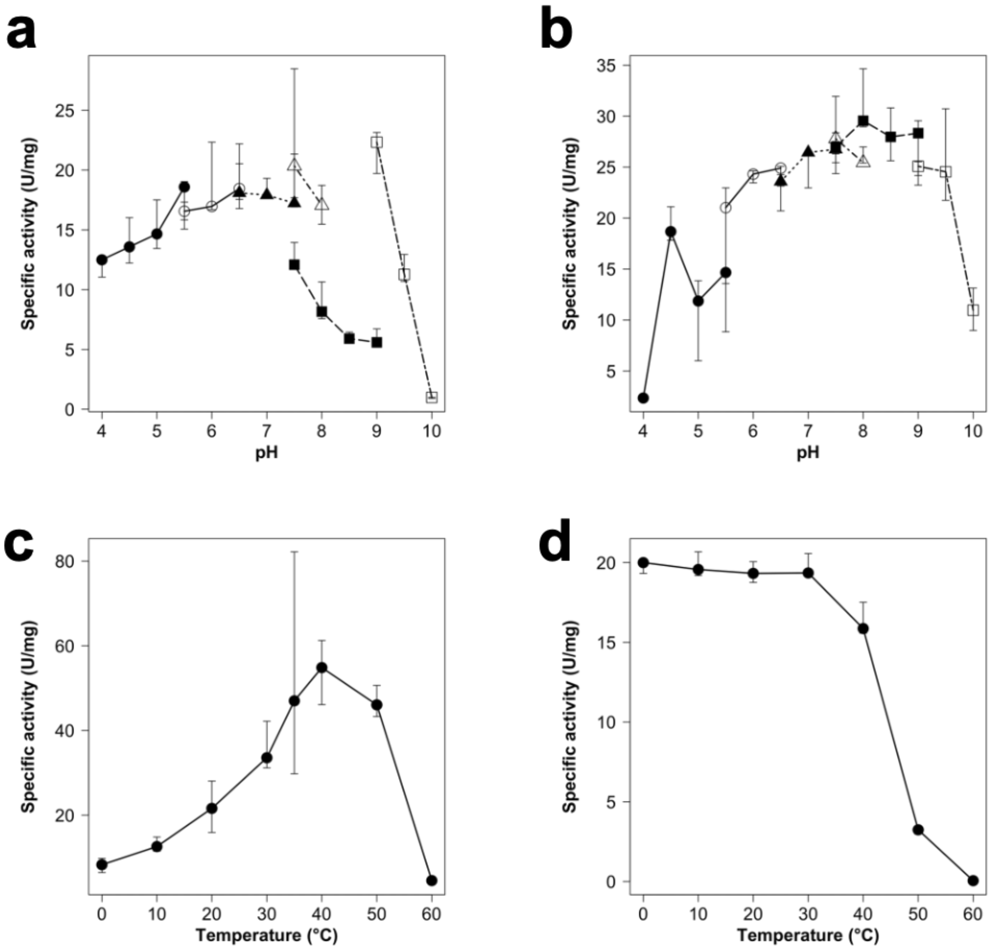

Supplementary Figure 3. pH and temperature profiles of Bxy_22780.

a–b, Optimum pH (**a**) and pH stability (**b**). Buffers used are sodium acetate (pH 4.0–5.5, closed circles), MES (pH 5.5–6.5, open circles), MOPS (pH 6.5–7.5, closed triangles), HEPES (pH 7.5–8.0, open triangles), Tris-HCl (pH 7.5–9.0, closed squares) and glycine (pH 9.0–10.0, open squares). **c–d**, Optimum temperature (**c**) and temperature stability (**d**). The quantitative data for hydrolytic activity were obtained from three independent experiments. Medians in triplicate experiments are shown as plots and the other data are shown as error bars.

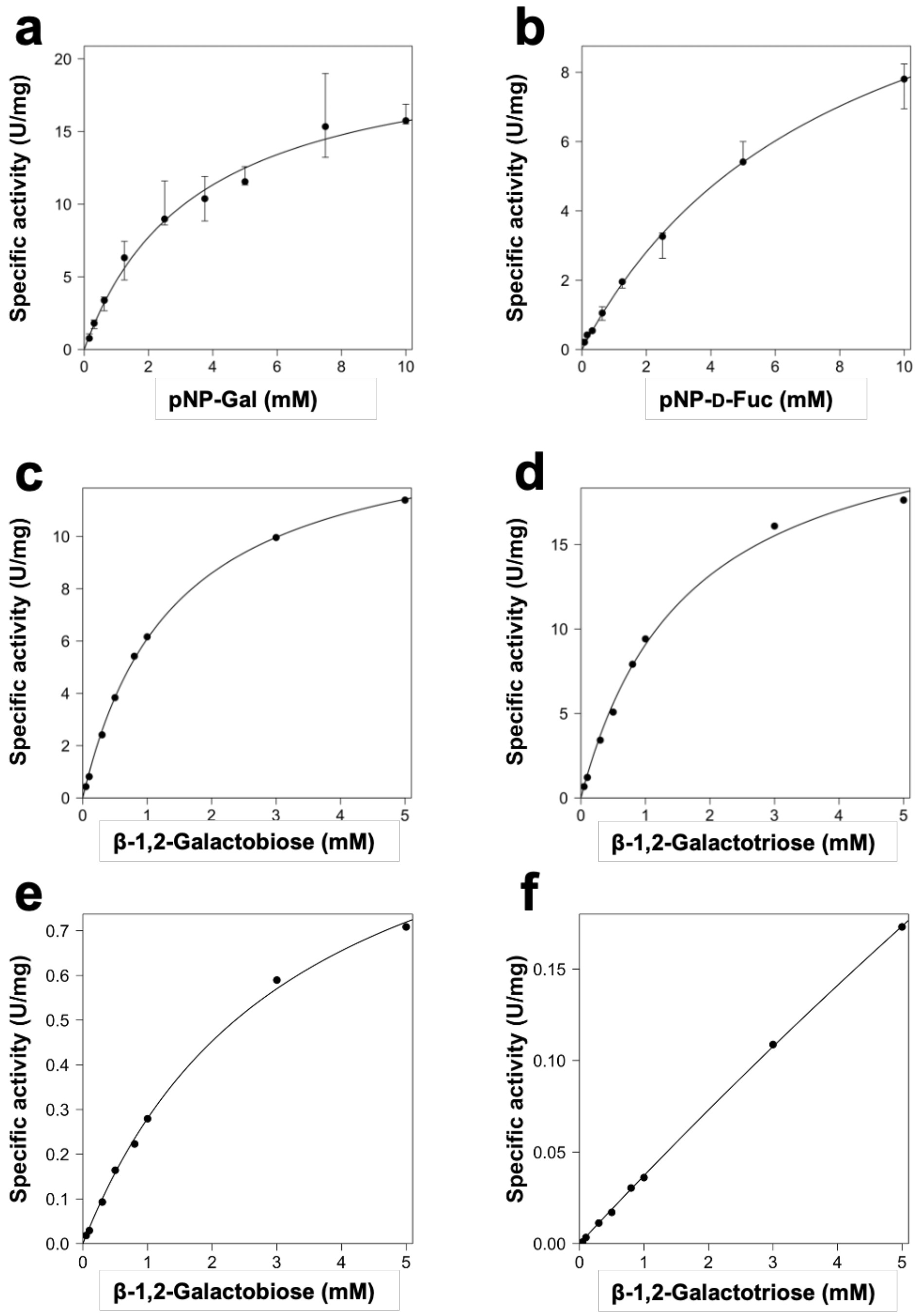

Supplementary Figure 4. Kinetic analysis of Bxy_22780.

Kinetic analysis for the wild-type (**a**–**d**) and W288A mutant (**e**–**f**). Experiments were performed triplicate (**a**–**b**) for pNP-sugars and once (**c**–**f**) for β-1,2-galactooligosaccharides. The quantitative data for hydrolytic activity on pNP-Gal and pNP-D-Fuc (**a**–**b**), and β-1,2-Gal_2 and β-1,2-Gal_3 (**c**–**f**) were obtained from three independent experiments and one experiment, respectively. Medians in triplicate experiments are shown as plots and the other data in the triplicate are used as error bars (**a**–**b**). Kinetic parameters are shown in Table 1.

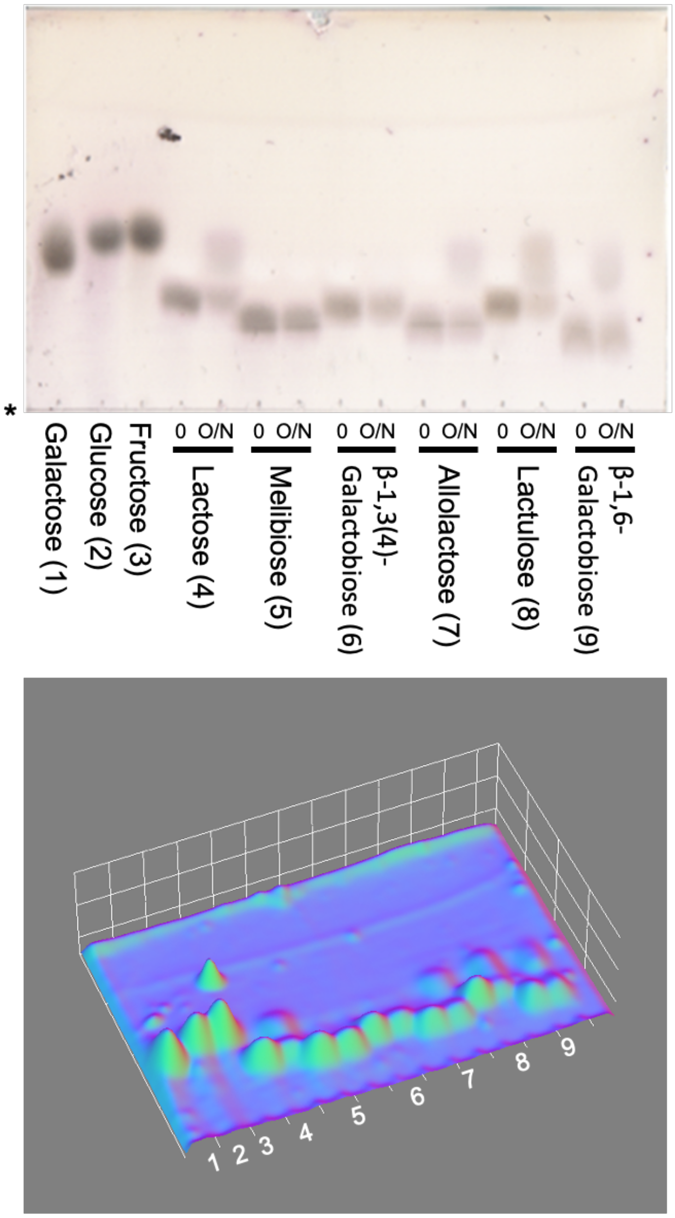

Supplementary Figure 5. Hydrolytic activity of Bxy_22780 toward galactosides.

(top) Each reaction was performed in the presence of 5 mM substrate and 0.1 mg/ml Bxy_22780 (wild-type). An asterisk represents an origin. Lanes Fructose, glucose and galactose are markers (1 μl of 10 mM solution). Reaction time are shown as “0” and “O/N” for 0 h and overnight, respectively. (bottom) Graphic visualization of the TLC plate by ImageJ [2]. The white numbers represent carbohydrates corresponding to the numbers in parentheses below the TLC plate.

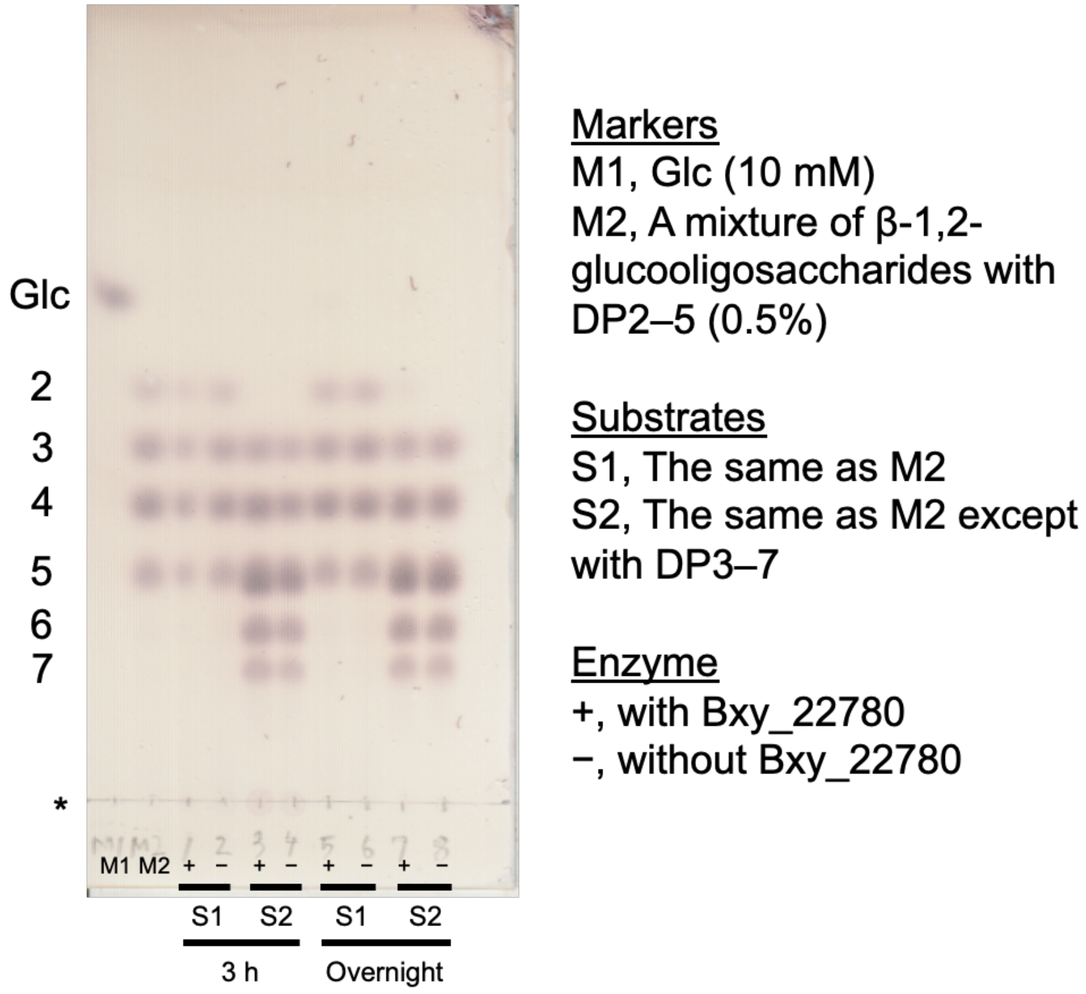

Supplementary Figure 6. Hydrolytic activity of Bxy_22780 toward β-1,2-glucooligosaccharides. Each reaction was performed in the presence of 0.5% substrate and 0.1 mg/ml Bxy_22780 (wild-type) for 3 h or overnight. An asterisk represents an origin. Numbers beside the TLC plate are DPs of β-1,2-glucooligosaccharides. The solution (1 μl) was spotted in each lane. The samples of 3 h reaction did not undergo heat treatment after the reaction.

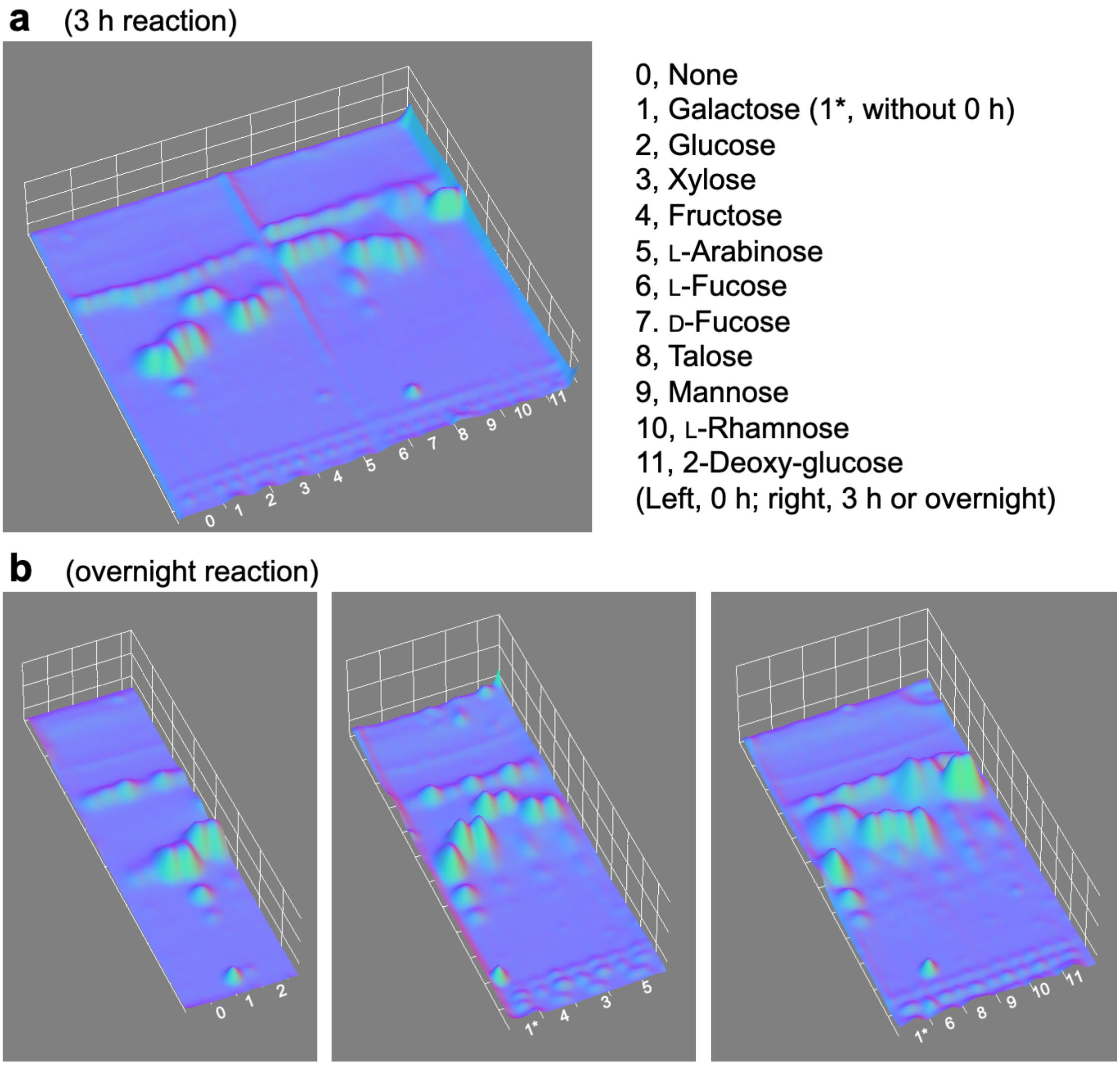

Supplementary Figure 7. Graphic visualization of the TLC plates for glycosynthase activity of Bxy_22780.

The TLC plates in Fig. 1a were visualized graphically by ImageJ [2].

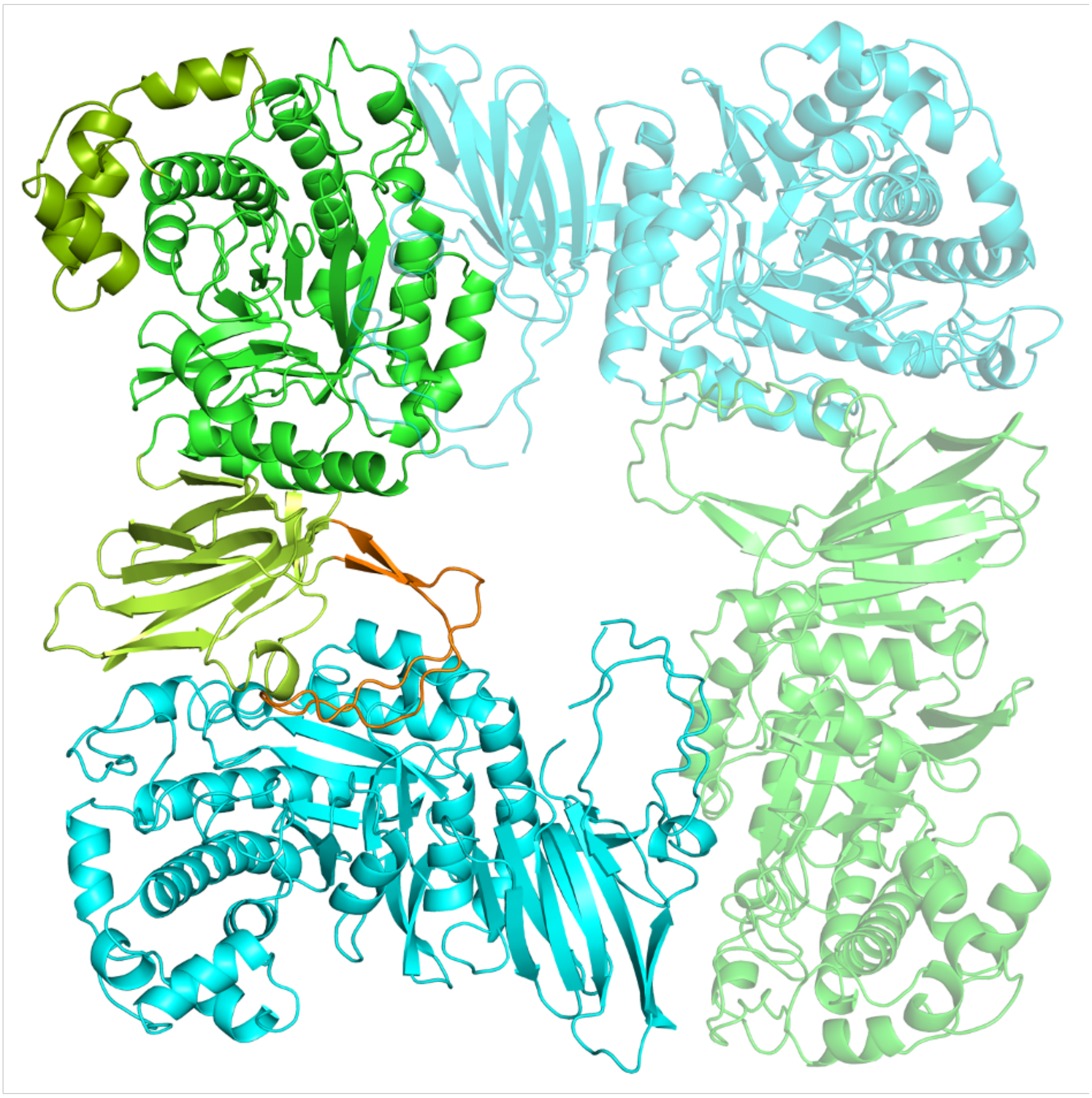

Supplementary Figure 8. Overall structure of Bxy_22780

PDB ID of the overall structure of E350G mutant is 8Z43. The two subunits in an asymmetric unit are fully colored while the other subunits in a symmetry mate are shown transparently. The two subunits in the asymmetric unit are colored in green and cyan basically. In the green colored subunit, a catalytic domain composed of TIM-barrel domain (residues 1–198 and 253–404) are left in green while an inserted region in the catalytic domain (residues 199–252), DUF5597 domain (residues 405–506 and 539–550), and an inserted region in DUF5597 domain are colored in dark green, light green and orange, respectively.

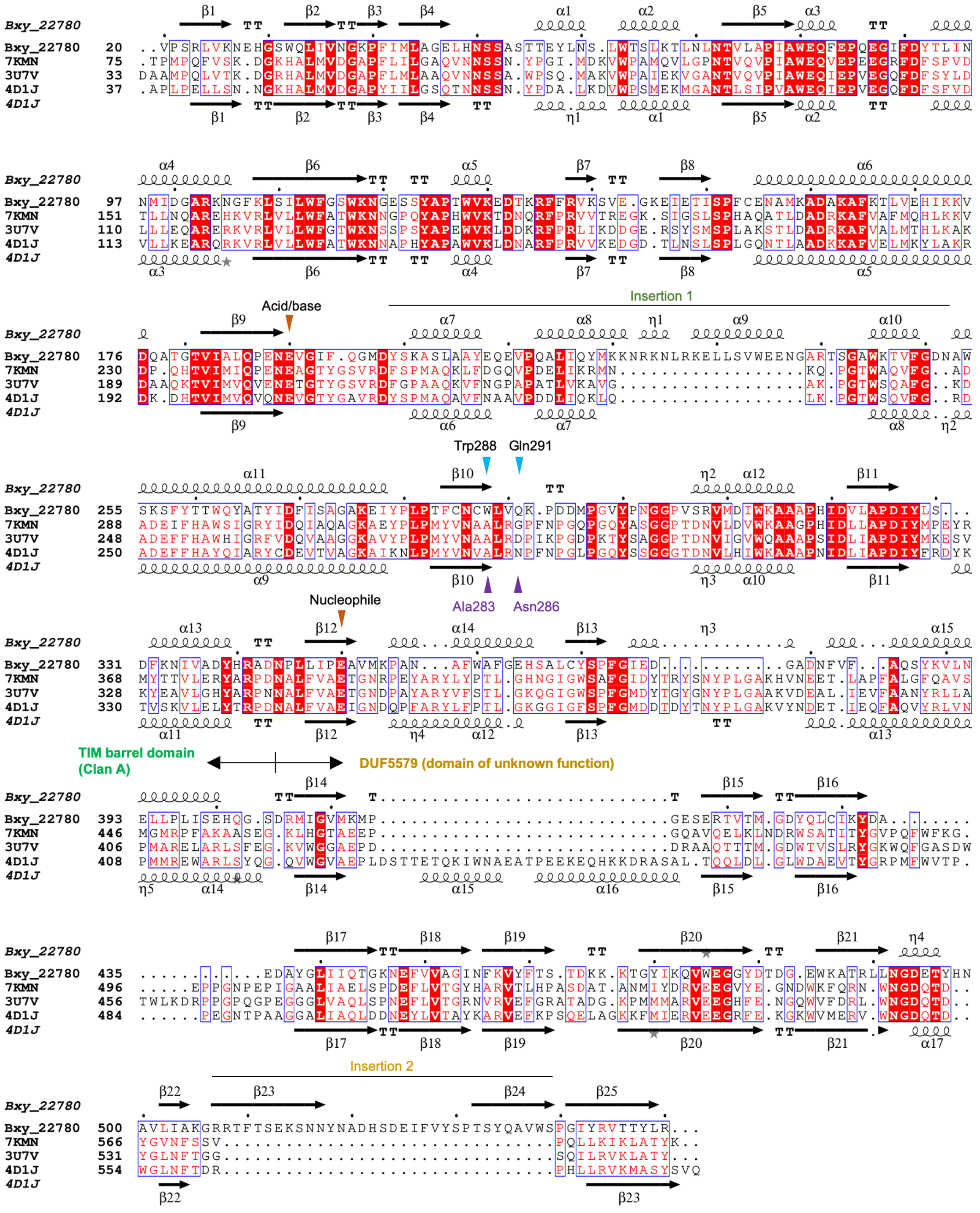

Supplementary Figure 9. Structure-based multiple sequence alignment

Multiple sequence alignment was performed using PDBeFold (https://www.ebi.ac.uk/msd-srv/ssm/) [3]. The alignment was visualized by ESPript ver3.0 (https://espript.ibcp.fr/ESPript/cgi-bin/ESPript.cgi) [4]. The constitution of the domains and insertion regions shown in Fig. S6 are added manually in the alignment. Catalytic residues (Glu190 and Glu350) and substrate recognition residues (Trp288 and Gln291) in Bxy_22780 are indicated with dark orange and cyan reversed triangles, respectively. The Ala283 and Asn286 in CjBgl35A corresponding to Trp288 and Gln291 are indicated with purple

triangles. The aligned amino acid sequences other than Bxy_22780 (GenBank accession NO., CBK67349.1) are shown as PDB IDs: 7KMN (*Xanthomonas citri* pv. *citri* str. 306, AAM36636.1), 3U7V (*Caulobacter vibrioides* CB15, AAK22773.1) and 4D1J (*Cellvibrio japonicus* Ueda107, ACE85180.1).

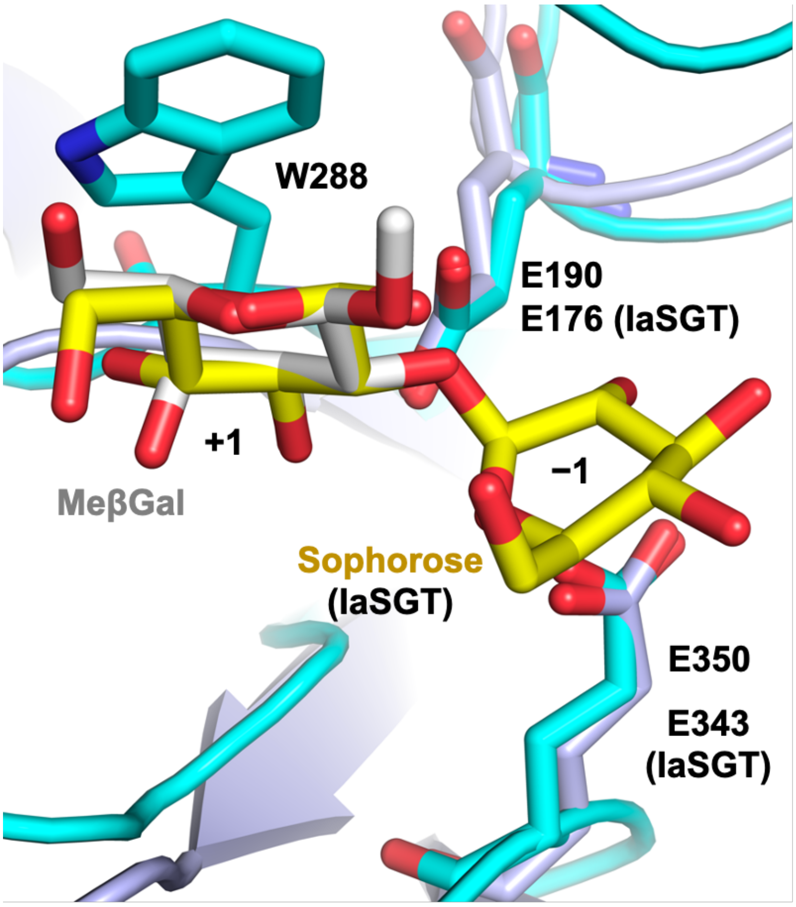

Supplementary Figure 10. Superimposition of Bxy_22780 and IaSGT

Complex structures of Bxy_22780 with MeβGal (PDB ID, 8Z48) and IaSGT with sophorose (PDB ID, 7VKY) were used for comparison. Bxy_22780 and IaSGT are shown in cyan and light purple, respectively. Catalytic residues and Trp288 in Bxy_22780 are shown as sticks. MeβGal and sophorose are shown as white and yellow sticks, respectively, with subsite numbers.

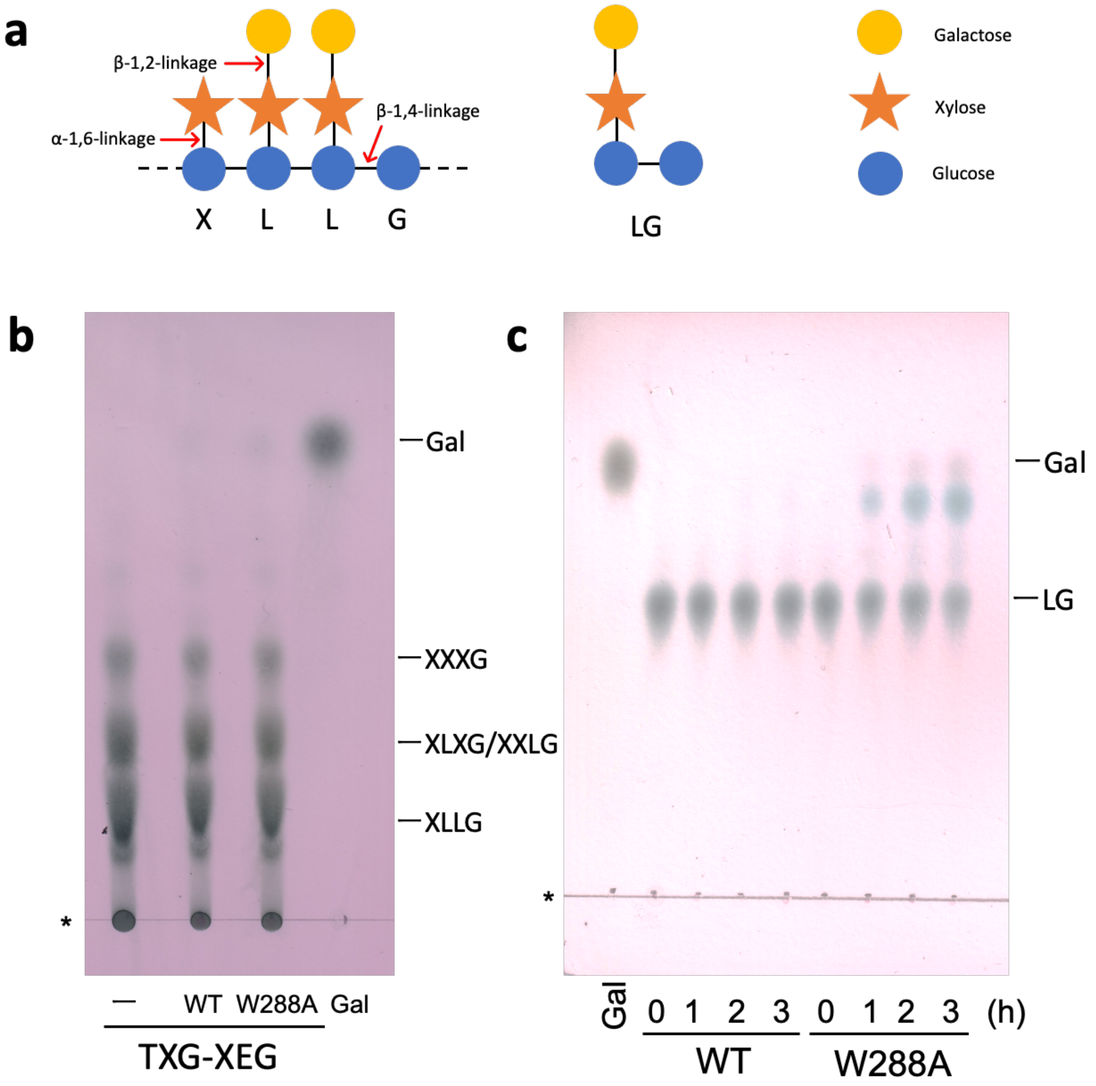

Supplementary Figure 11. Activity toward xyloglucan associated substrates

a, Schematic representation of tamarind-xyloglucan. Representation of side chain patterns in xyloglucan is used in **b**, **c**: G, no side chain; X, with xylose as a side chain; L, galactosyl xylose as a side chain. Symbols of monosaccharides illustrated based on Symbol Nomenclature for Glycans (https://www.ncbi.nlm.nih.gov/glycans/snfg.html). **b** and **c**, Action pattern analysis toward TXG-XEG (**b**) and LG (**c**) by TLC. Lane Gal, 10 mM Gal (1 μl) was spotted. Lane −, TXG-XEG without enzyme. Asterisks represent the origin on the TLC plates.

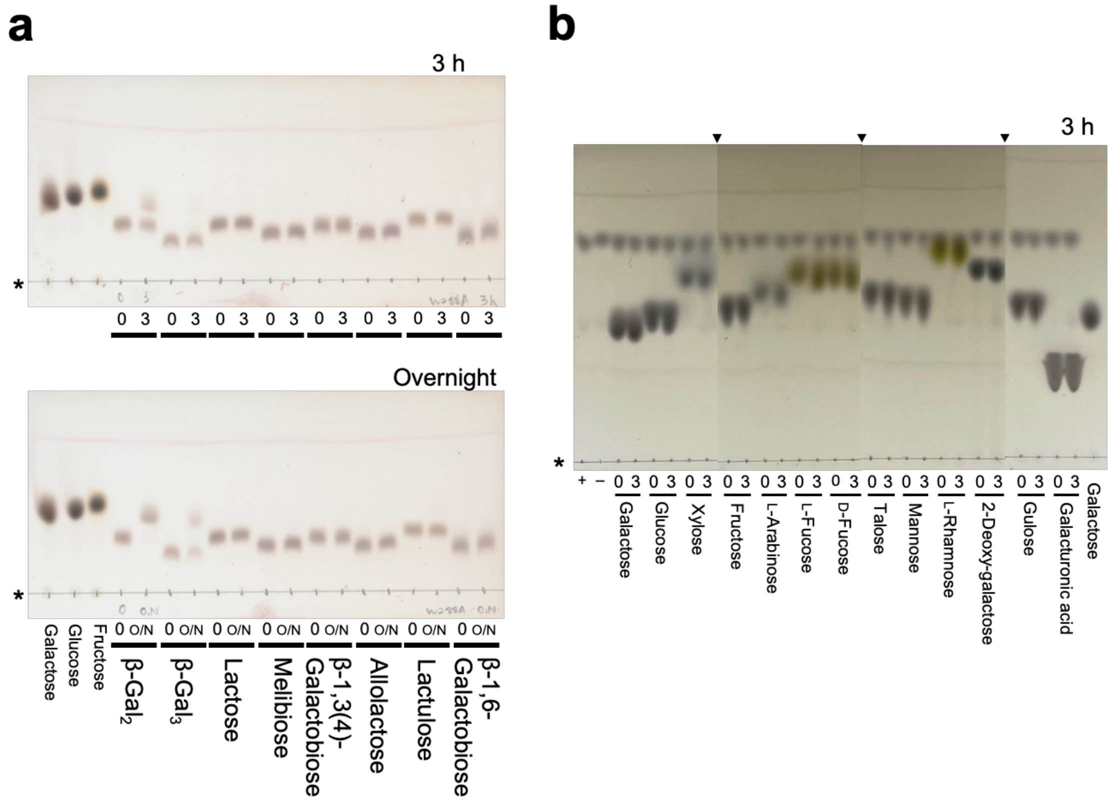

Supplementary Figure 12. Substrate specificity of W288A mutant

a, Hydrolytic activity of W288A mutant. **b**, Glycosynthase activity of W288A/E350G mutant. Lanes without bars are markers (1 µl of 10 mM solution). Lanes indicated with + and – represent αGalF was incubated with and without W288A/E350G mutant, respectively. Reaction times are shown above the bars and the plates. Reverse triangles above the plates represent the boundaries of TLC plates.

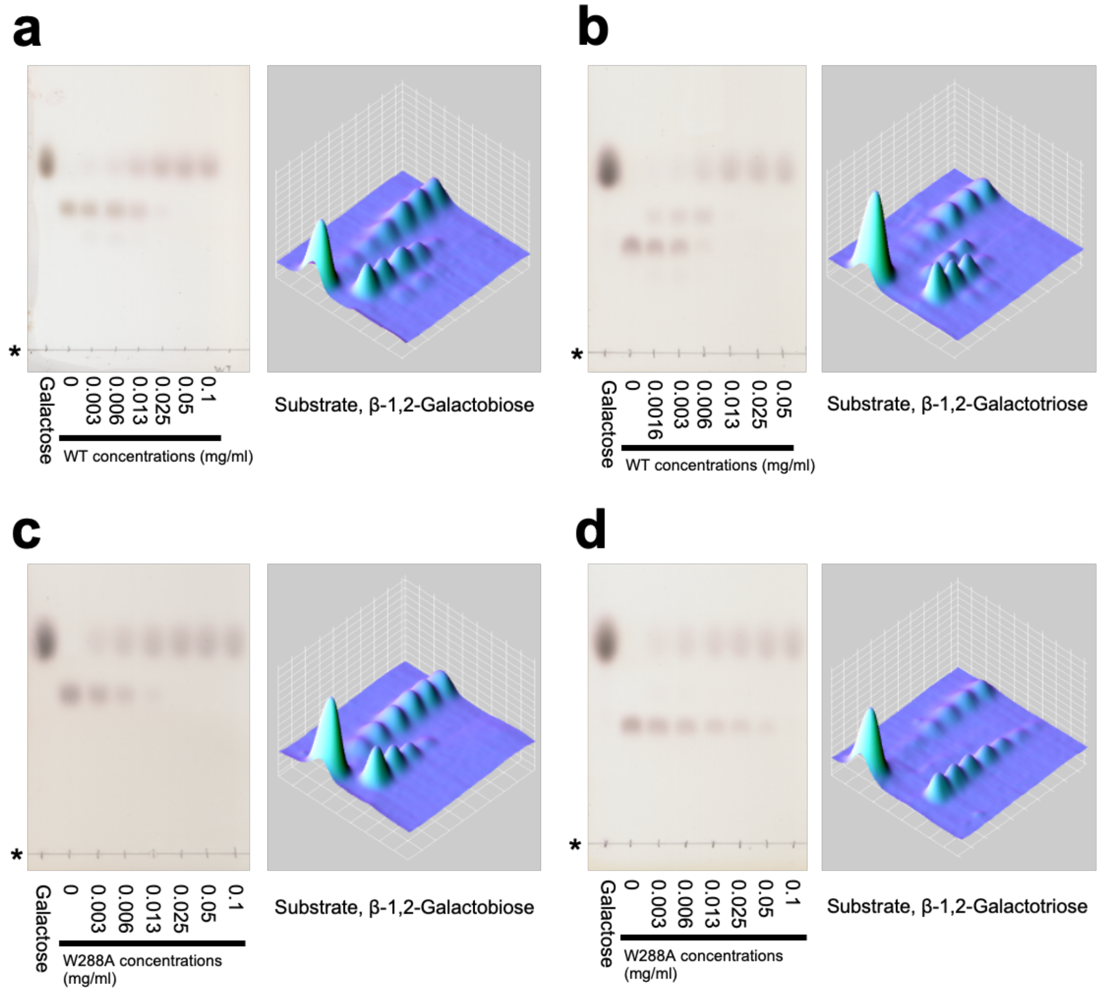

Supplementary Figure 13. Transglycosylation activity toward β-1,2-galactooligosaccharides

(Left) Action patterns shown by TLC plates. Lane Galactose, 1 µl of 10 mM galactose was spotted. Asterisks represent the origins. (Right) Graphic visualization of the TLC plates by ImageJ [2].

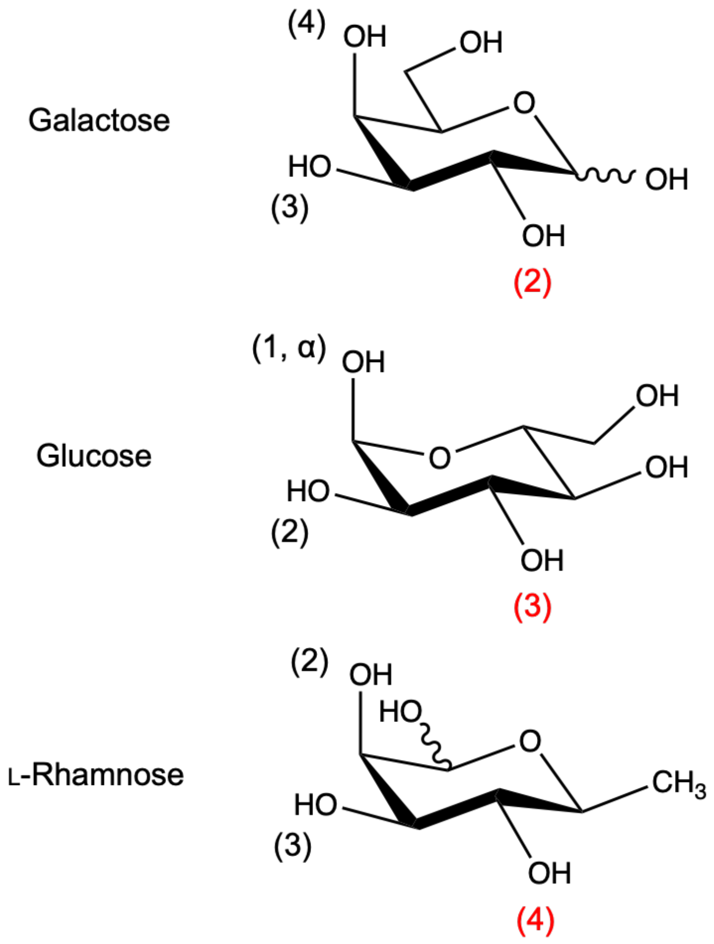

Supplementary Figure 14. Comparison of conformations between galactose, glucose and L-rhamnose.

Orientations of glucose and L-rhamnose were changed so that orientations of three hydroxy groups in the two monosaccharides correspond to those of 2-, 3- and 4-hydroxy groups in galactose. Numbers in parentheses represent the positions of hydroxy groups. α in parenthesis is an orientation of anomeric hydroxy group. Red numbers represent a linkage position in galactose at subsite +1 and presumed linkage positions in glucose and L-rhamnose.

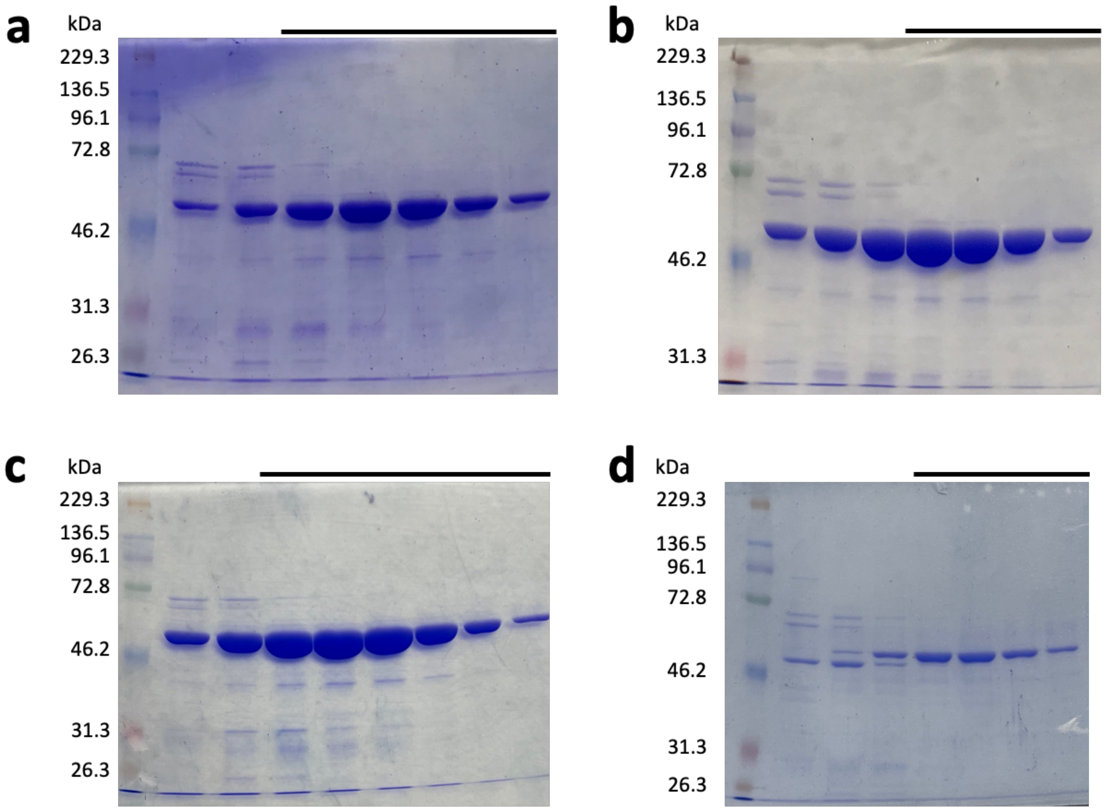

Supplementary Figure 15. SDS-PAGE of Bxy_22780

Fractions obtained using Nickel affinity column for purification of the wild-type (**a**), E350G mutant (**b**), W288A mutant (**c**) and W288A/E350G mutant (**d**). Bars above the gels represent the fractions collected for further use. Left lanes are marker proteins.

Supplementary Table 1. Primers used in this study

	Sequence (5′ to 3′)	
Subcloning of the whole gene in databases		
Fw	TTTCATATGGGAACTTCAATGATGAATG	NdeI
Rv	TATGAATAAGCTGCTCGAGCCGTAAATAGGTTGTC	XhoI
Deletion of the N-terminal signal peptide		
Fw	ACATATGCGCCCCCCAATGTATACT	
Rv	GGGGGGCGCATATGTATATCTCCTTC	
Mutation of E350G		
Fw	ATTCCCGGTGCAGTGATGAAACCGGCC	
Rv	CACTGCACCGGGAATCAATAACGGATT	
Mutation of W288A		
Fw	AACTGTGCACTGGTGCAAAAGCCTGAT	
Rv	CACCAGTGCACAGTTGCAGAAGGTAGG	
For assay		
TM1867 (L-lactate dehydrogenase)		
Fw	GTGATTCATATGAAAATAGGTATCG	NdeI
Rv	CTCTACCTCGAGTTAACCGCTGGTG	XhoI
TM1190 (galactokinase)		
Fw	TTTAAGAAGGAGATATACATATGAAAGTGAAGGCACCAGGAAG	
Rv	GTGGTGGTGGTGGTGGTGCTCGAGGATTTTTTGAACACCGTCTGAAC	

Restriction sites are indicated with underlines and restriction enzymes acting on the underlined sequences are shown beside the sequences. Fw, forward primer; Rv, reverse primer.

References

1. Kumar, S., Stecher, G., Li, M., Knyaz, C. & Tamura, K. MEGA X: Molecular evolutionary genetics analysis across computing platforms. *Mol Biol Evol* **35**, 1547–1549 (2018).
2. Schneider, C. A., Rasband, W. S. & Eliceiri, K. W. NIH Image to ImageJ: 25 years of image analysis. *Nat Methods* **9**, 671–675 (2012).
3. Krissinel, E. & Henrick, K. Secondary-structure matching (SSM), a new tool for fast protein structure alignment in three dimensions. *Acta Crystallogr D Biol Crystallogr* **60**, 2256–2268 (2004).
4. Robert, X. & Gouet, P. Deciphering key features in protein structures with the new ENDscript server. *Nucleic Acids Res* **42**, 320–324 (2014).
