# Supplementary figures and images for: Structure and function of a β-1,2-galactosidase from Bacteroides xylanisolvens, an intestinal bacterium
Source: Commun Biol. 2025 Jan 16;8:66. doi: 10.1038/s42003-025-07494-1 (PMC11739564; doi:10.1038/s42003-025-07494-1)

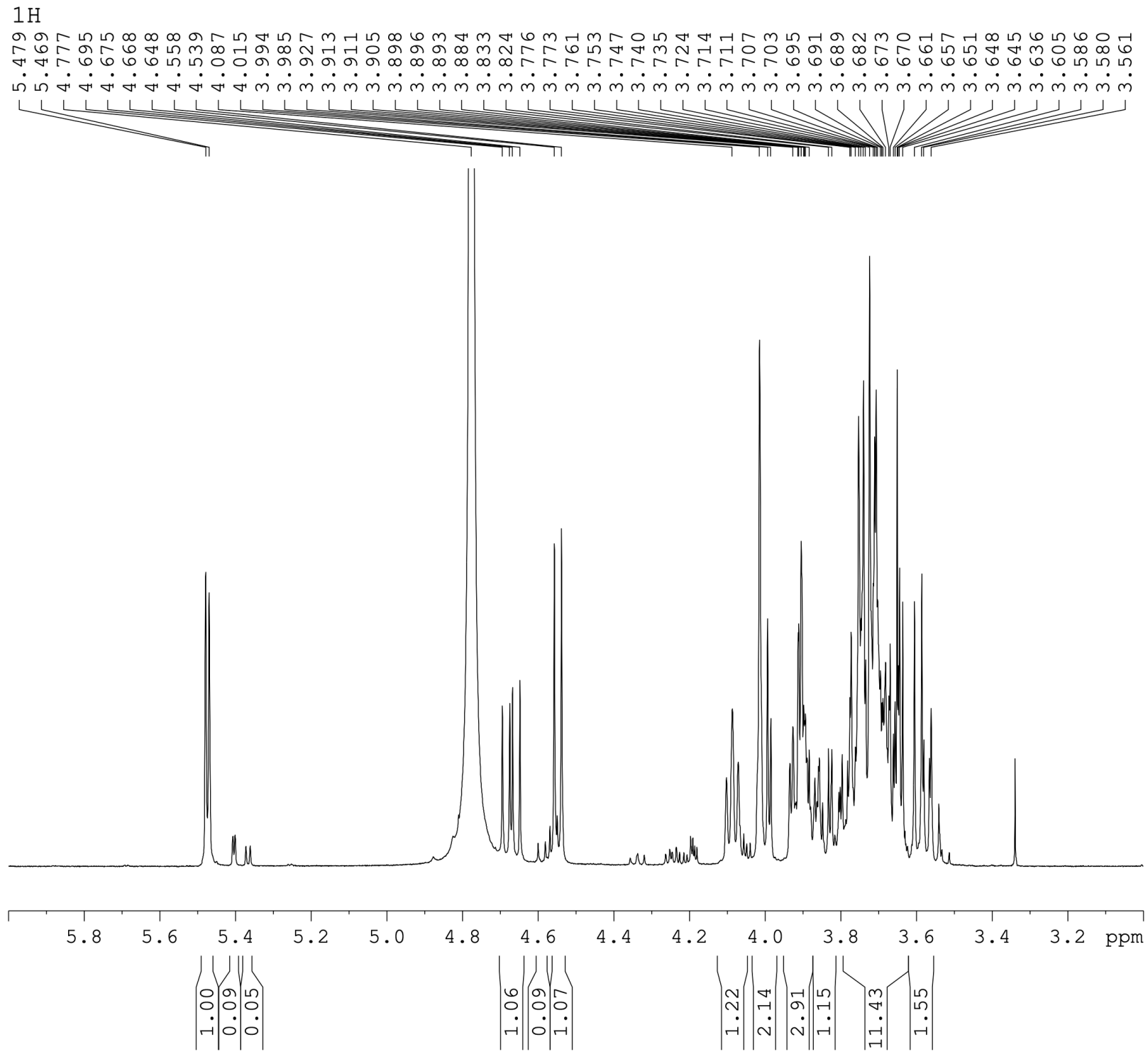

<sup>13</sup>C

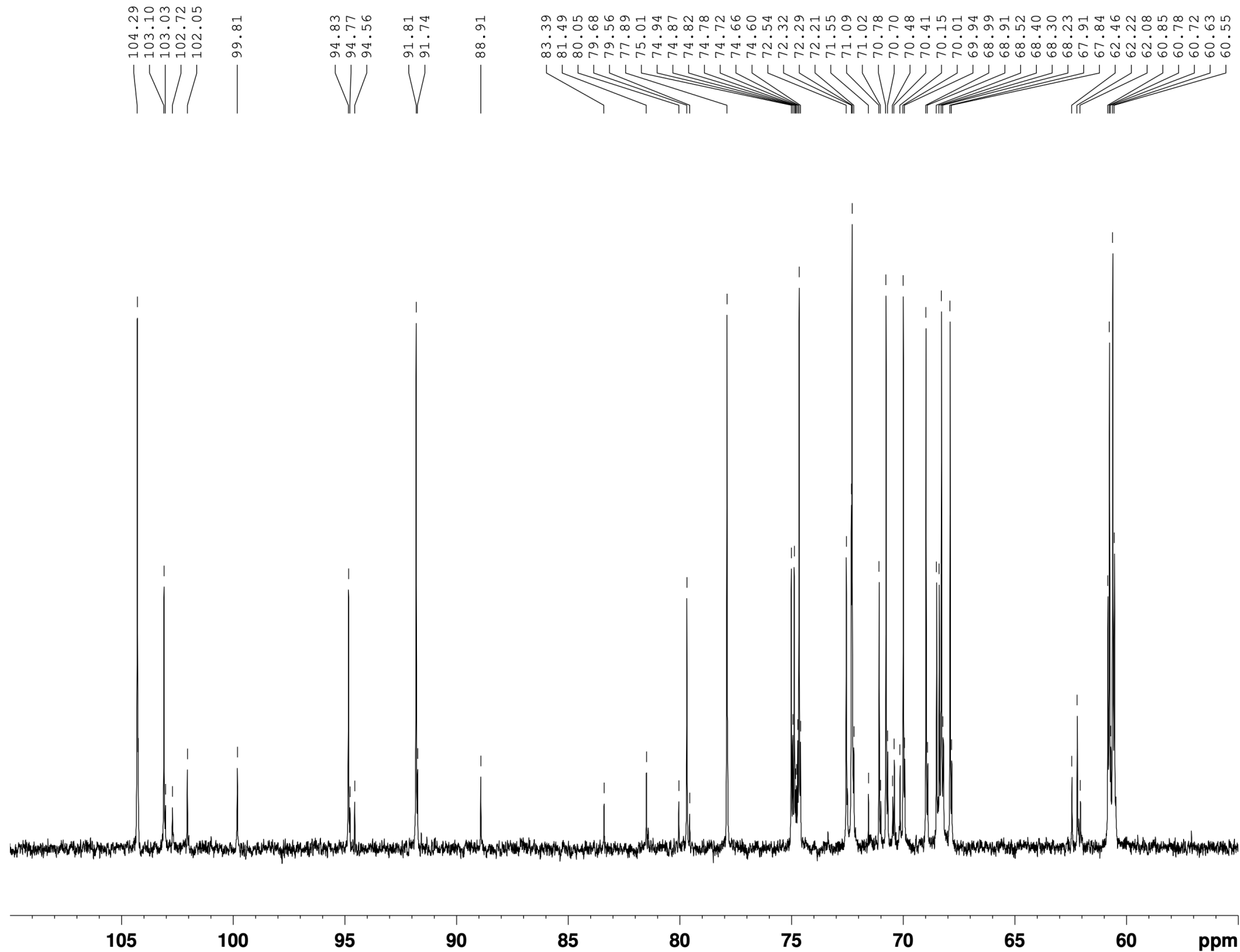

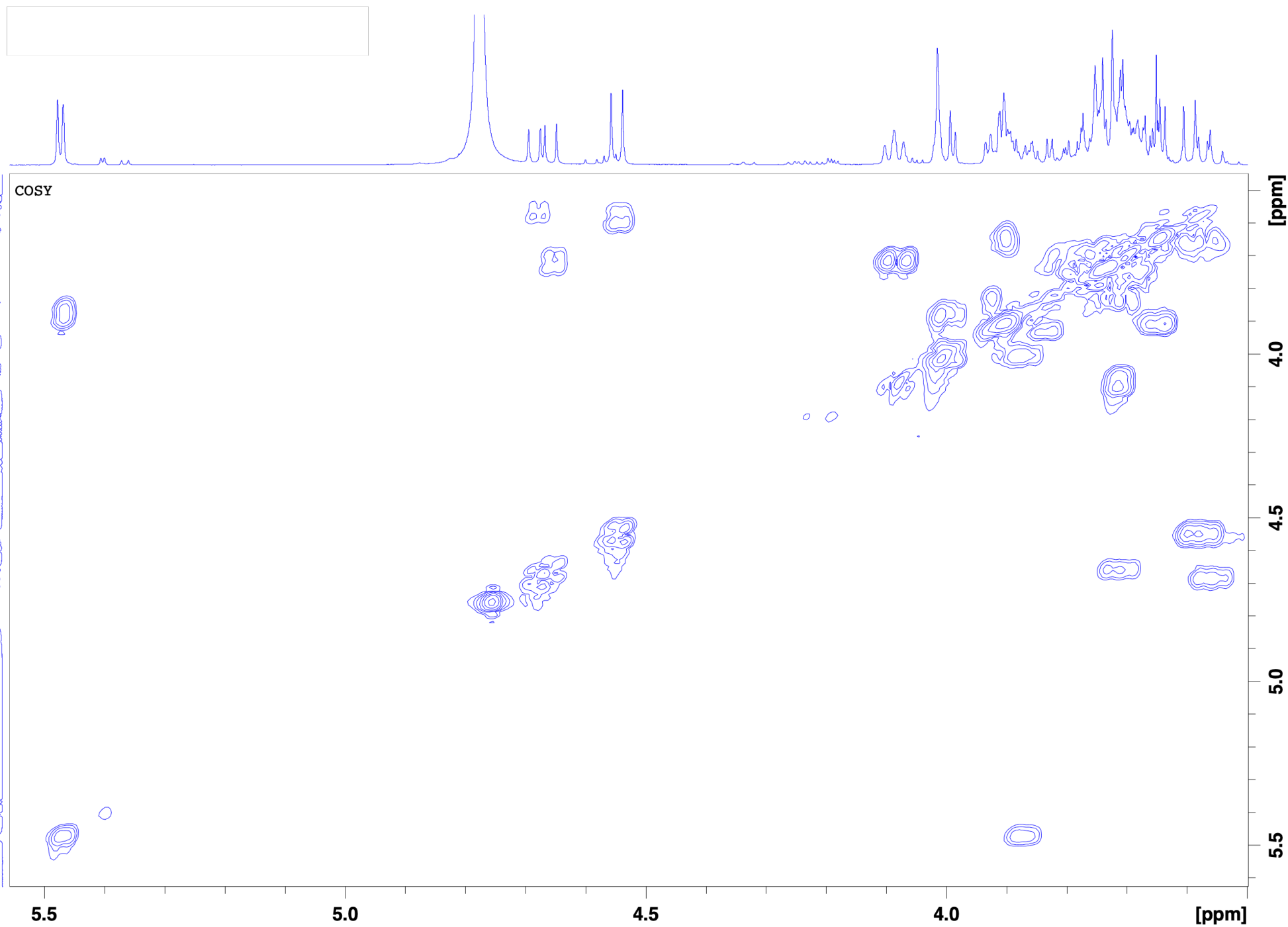

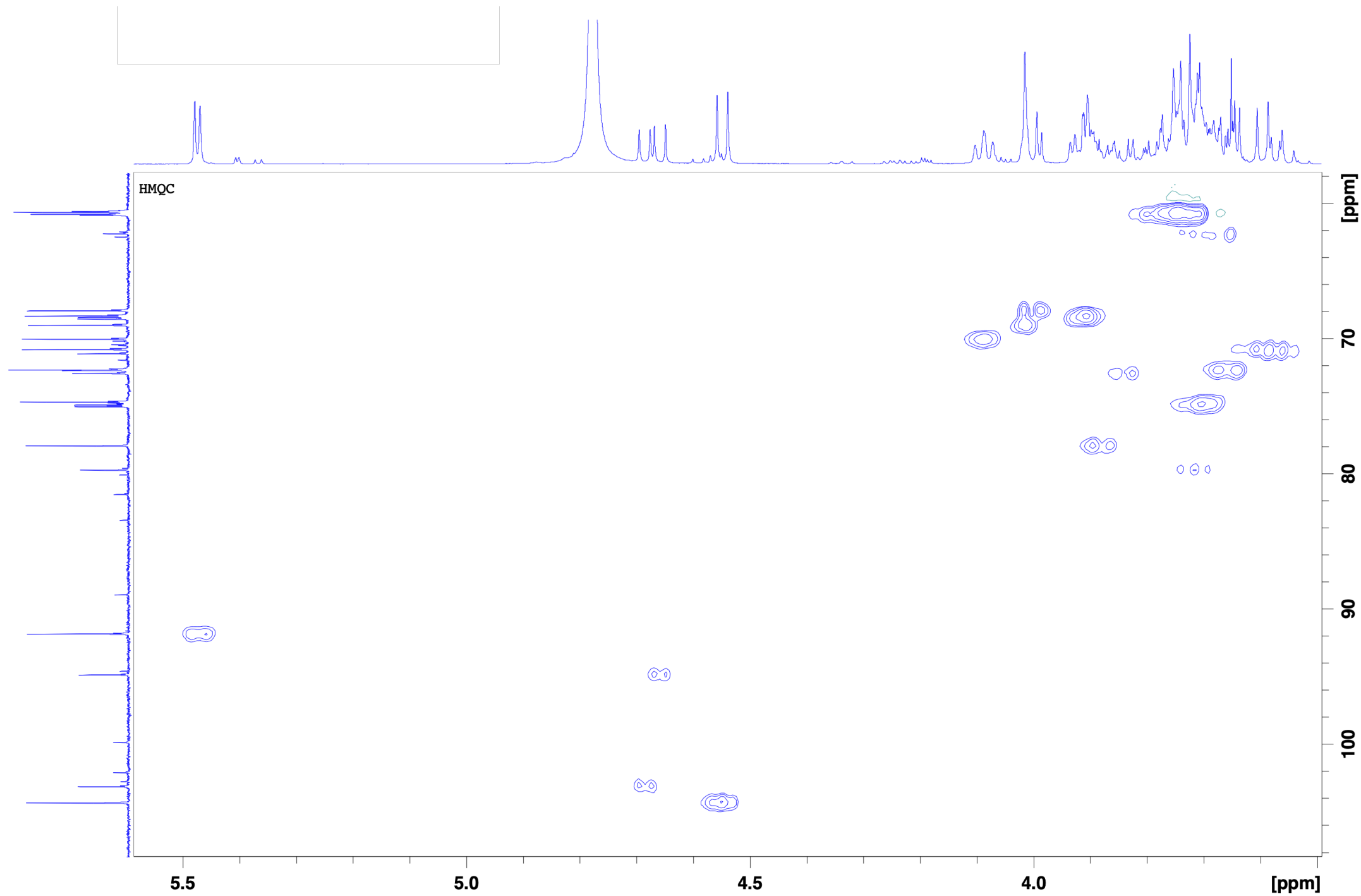

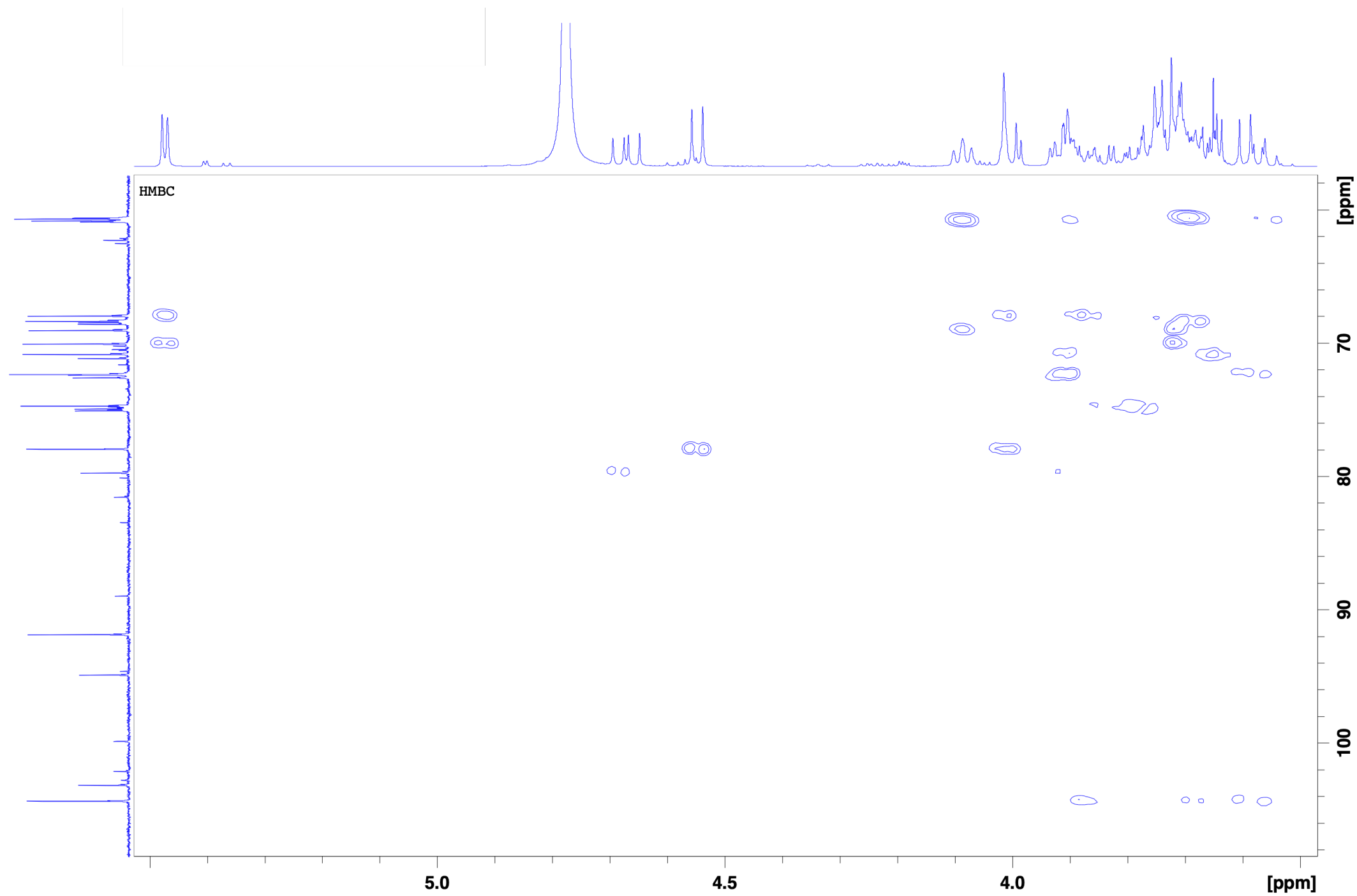

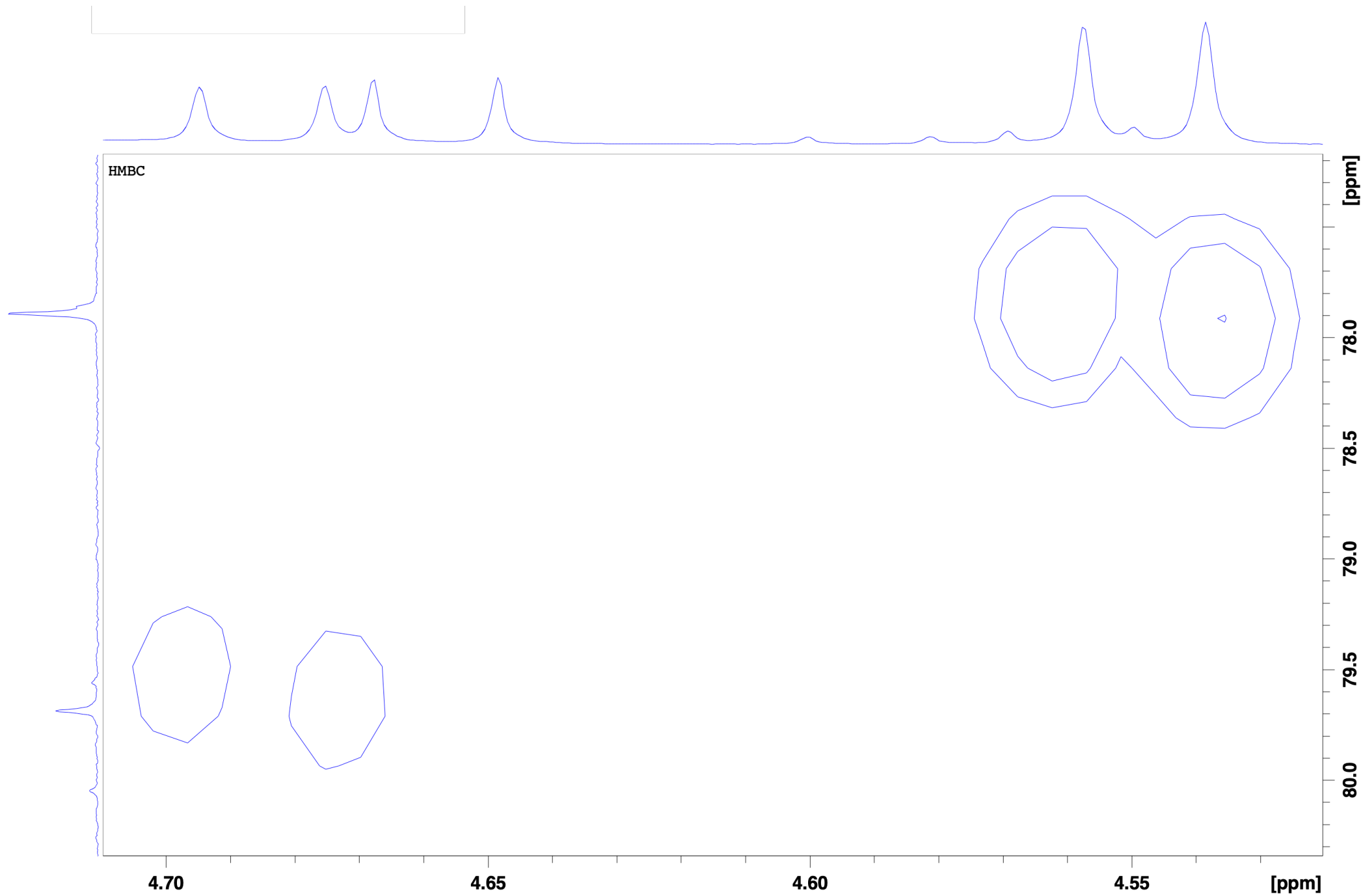

Supplement: Supplementary file 2 — Supplementary Data 1 [file 42003_2025_7494_MOESM2_ESM.pdf]
