# Supplementary material for: Structure and function of a β-1,2-galactosidase from Bacteroides xylanisolvens, an intestinal bacterium
Source: Commun Biol. 2025 Jan 16;8:66. doi: 10.1038/s42003-025-07494-1 (PMC11739564; doi:10.1038/s42003-025-07494-1)
Supplement: Supplementary file 3 — Supplementary Data 2 [file 42003_2025_7494_MOESM3_ESM.pdf]

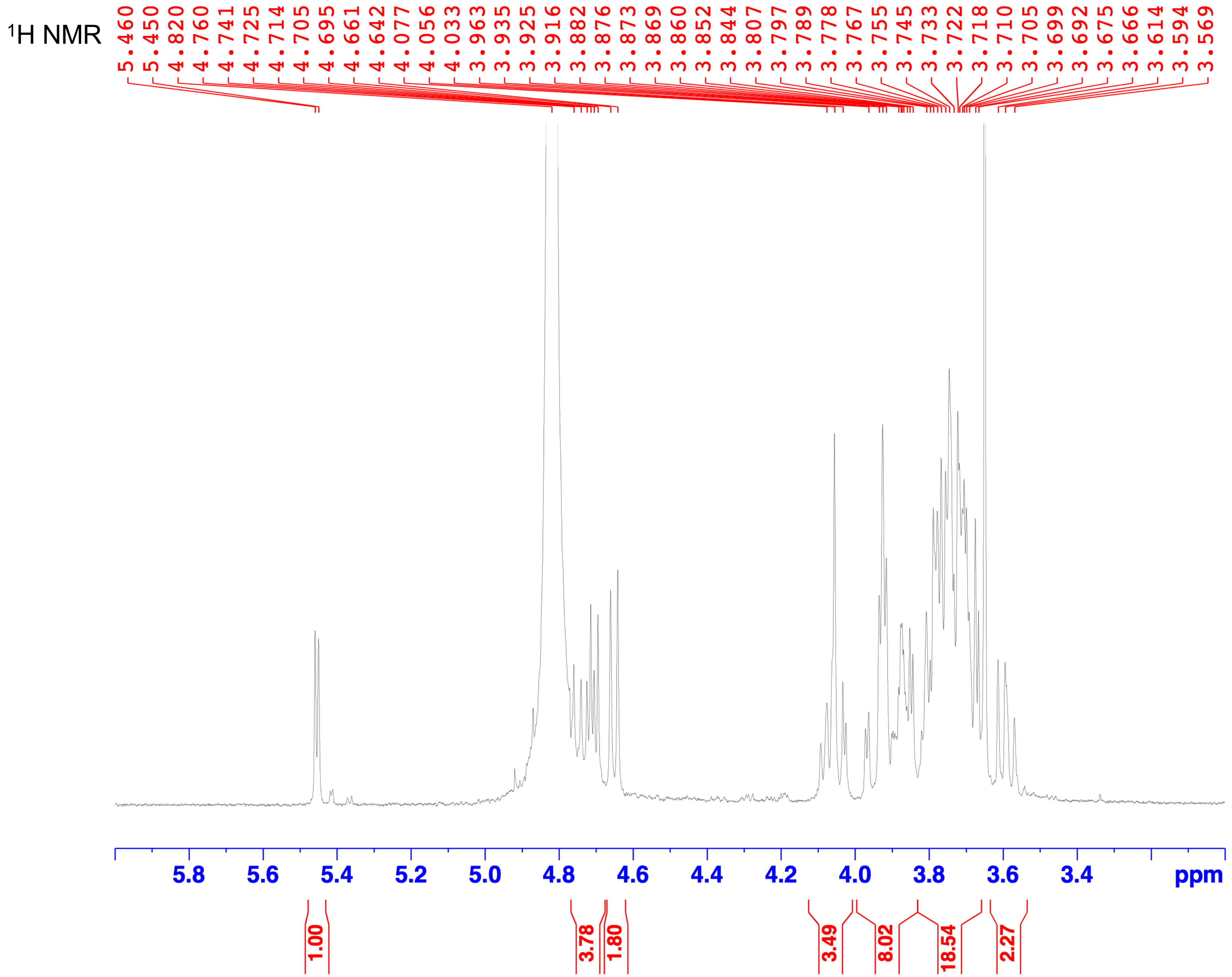

1H NMR
5.460
5.450
4.820
4.760
4.741
4.725
4.714
4.705
4.695
4.661
4.642
4.077
4.056
4.033
3.963
3.935
3.925
3.916
3.882
3.876
3.873
3.869
3.860
3.852
3.844
3.807
3.797
3.789
3.778
3.767
3.755
3.745
3.733
3.722
3.718
3.710
3.705
3.699
3.692
3.675
3.666
3.614
3.594
3.569
5.8
5.6
5.4
5.2
5.0
4.8
4.6
4.4
4.2
4.0
3.8
3.6
3.4
ppm
1.00
3.78
1.80
3.49
8.02
18.54
2.27

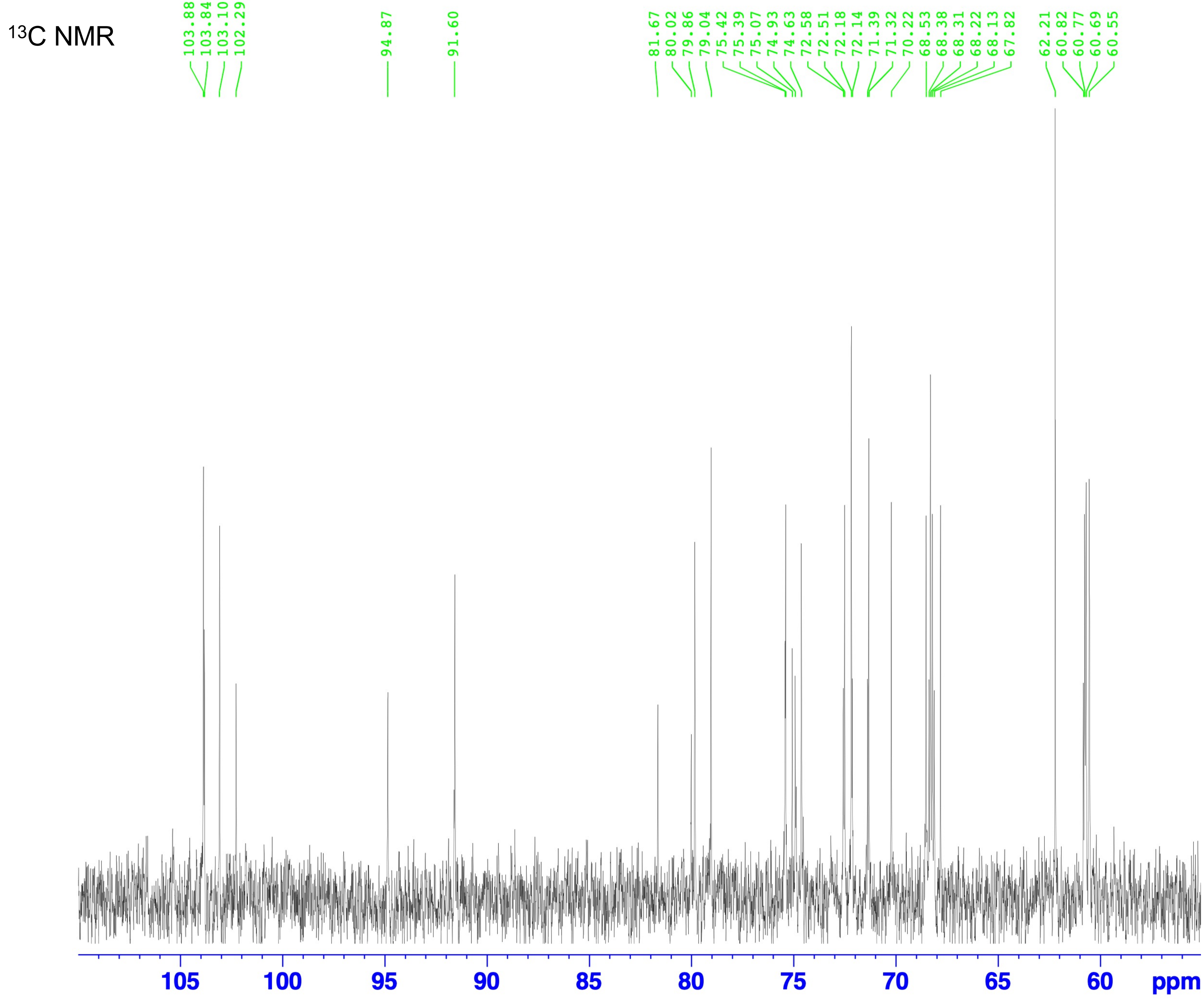

13C NMR
103.88
103.84
103.10
102.29
94.87
91.60
81.67
80.02
79.86
79.04
75.42
75.39
75.07
74.93
74.63
72.58
72.51
72.18
72.14
71.39
71.32
70.22
68.53
68.38
68.31
68.22
68.13
67.82
62.21
60.82
60.77
60.69
60.55
105
100
95
90
85
80
75
70
65
60
ppm

^{13}C NMR
(95–105 ppm)

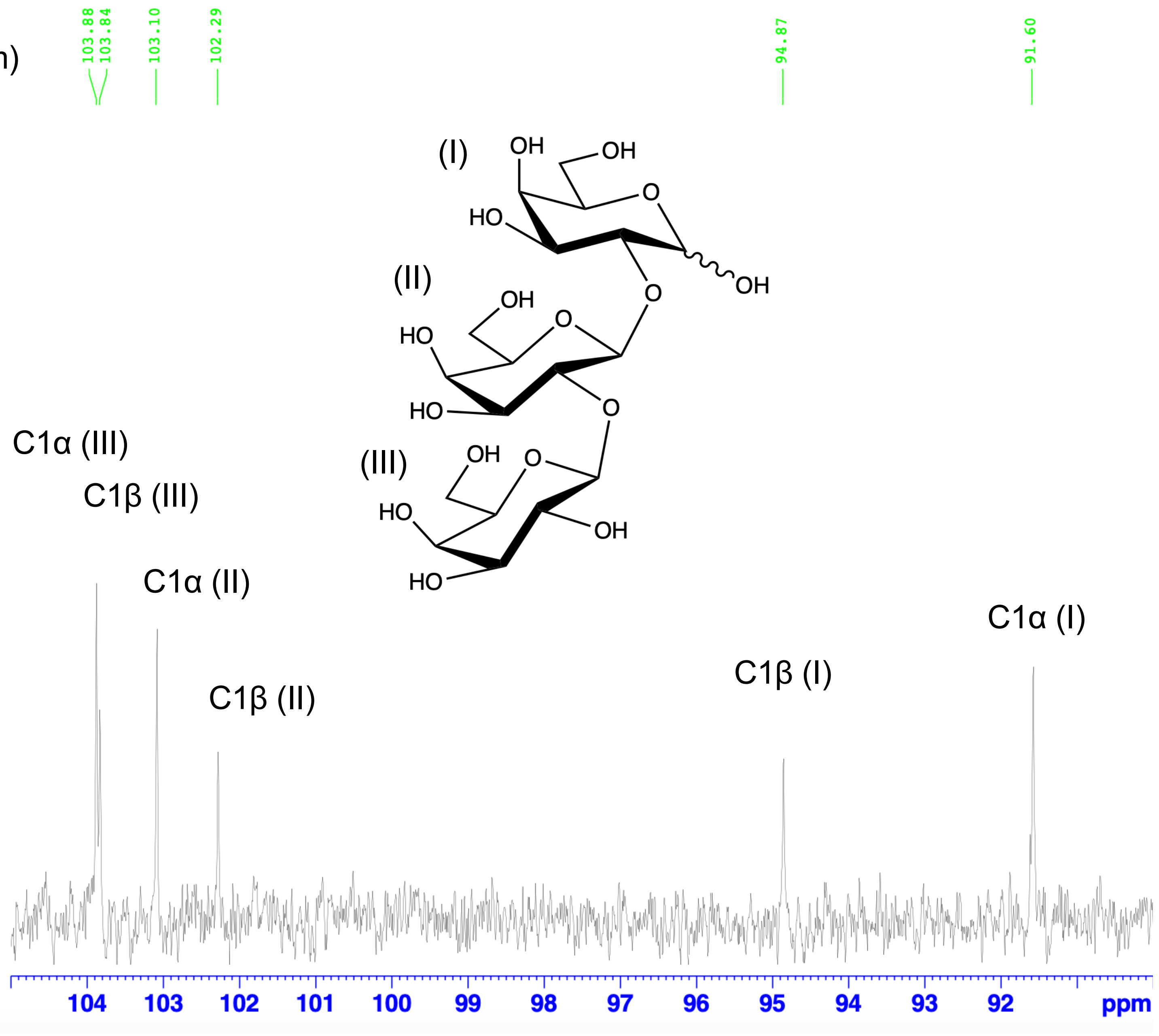

* Assignment is based on ratio of α and β anomers, and general patterns of chemical shifts in galactosides.
