# Supplementary material for: Structure and function of a β-1,2-galactosidase from Bacteroides xylanisolvens, an intestinal bacterium
Source: Commun Biol. 2025 Jan 16;8:66. doi: 10.1038/s42003-025-07494-1 (PMC11739564; doi:10.1038/s42003-025-07494-1)
Supplement: Supplementary file 4 — Supplementary Data 3 [file 42003_2025_7494_MOESM4_ESM.pdf]

Sample Name: KK2401

Date: 2024/10/15

Instrument: Sciex X500R QTOF

Ionization: ESI (Positive mode)

Solvent: H_2O containing 5 mM ammonium acetate

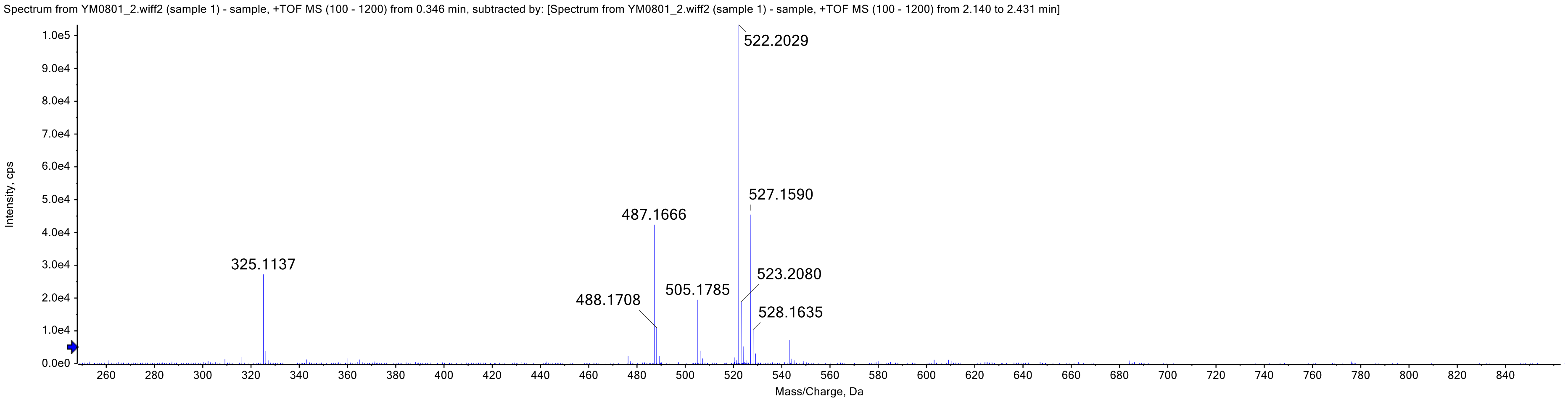

Formula $[M+NH_4]^+$	Theoretical *m/z*	Found *m/z*	RDB	Delta *m/z* (ppm)	Resolution
$C_{18}H_{36}NO_{16}$	522.2029	522.2029	2.0	0.1	30139
