# Supplementary material for: Structure and function of a β-1,2-galactosidase from Bacteroides xylanisolvens, an intestinal bacterium
Source: Commun Biol. 2025 Jan 16;8:66. doi: 10.1038/s42003-025-07494-1 (PMC11739564; doi:10.1038/s42003-025-07494-1)
Supplement: Supplementary file 6 — Description of Additional Supplementary Materials [file 42003_2025_7494_MOESM6_ESM.pdf]

Description of Additional Supplementary Files

File name: Supplementary Data 1

Description: NMR data of β-1,2-galactobiose

File name: Supplementary Data 2

Description: NMR data of β-1,2-galactotriose

File name: Supplementary Data 3

Description: ESI-MS data of β-1,2-galactotriose

File name: Supplementary Data 4

Description: Numerical source data for graphs
